# Association between maternal cardiometabolic markers and fetal growth in non-complicated pregnancies: a secondary analysis of the PRINCESA cohort

**DOI:** 10.1038/s41598-024-59940-5

**Published:** 2024-04-20

**Authors:** Isabel Omaña-Guzmán, Luis Ortiz-Hernández, Monica Ancira-Moreno, Myrna Godines-Enriquez, Marie O’Neill, Felipe Vadillo-Ortega

**Affiliations:** 1 Doctorado en Ciencias Biológicas y de la Salud, Universidad Autónoma Metropolitana, Mexico City, Mexico; 2 Unidad de Vinculación Científica de la Facultad de Medicina, Instituto Nacional de Medicina Genómica, Universidad Nacional Autónoma de México, Periférico Sur 4809, Arenal Tepepan, 14610 Mexico City, CDMX Mexico; 3 Pediatric Obesity Clinic and Wellness Unit, Hospital General de México “Dr. Eduardo Liceaga”, Mexico City, Mexico; 4 Departamento de Atención a la Salud, Universidad Autónoma Metropolitana, Mexico City, Mexico; 5 Departamento de Salud, Universidad Iberoamericana, Mexico City, Mexico; 6 Instituto Nacional de Perinatología Isidro Espinosa de los Reyes, Mexico City, Mexico; 7 Epidemiology and Environmental Health Sciences, School of Public Health, University of Michigan, Ann Arbor, MI USA

**Keywords:** Birth weight, Fetal growth, Fetal weight, Longitudinal study, Maternal cardiometabolic risk, Mexico, Pregnancy, Biomarkers, Epidemiology

## Abstract

The objective of this study was to evaluate the association of maternal cardiometabolic markers trajectories (glucose, triglycerides (TG), total cholesterol, systolic blood pressure (SBP) and diastolic blood pressure (DBP)) with estimated fetal weight trajectories and birth weight in Mexican pregnant women without medical complications. Cardiometabolic marker trajectories were characterized using group-based trajectory models. Mixed-effect and linear regression models were estimated to assess the association of maternal trajectories with estimated fetal weight and birth weight. The final sample comprised 606 mother–child dyads. Two trajectory groups of maternal cardiometabolic risk indicators during pregnancy were identified (high and low). Fetuses from women with higher values of TG had higher weight gain during pregnancy ($\hat{\beta }$ = 24.00 g; 95%CI: 12.9, 35.3), were heavier at the sixth month ($\widehat{\beta }$=48.24 g; 95%CI: 7.2, 89.7) and had higher birth weight ($\widehat{\beta }$= 89.08 g; 95%CI: 20.8, 157.4) than fetuses in the low values trajectory. Fetuses from mothers with high SBP and DBP had less weight in the sixth month of pregnancy ($\widehat{\beta }$= − 42.4 g; 95%CI: − 82.7, − 2.1 and $\widehat{\beta }$= − 50.35 g; 95%CI: − 94.2, − 6.4), and a higher DBP trajectory was associated with lower birth weight ($\widehat{\beta }$= − 101.48 g; 95%CI: − 176.5, − 26.4). In conclusion, a longitudinal exposition to high values of TG and BP was associated with potentially adverse effects on fetal growth. These findings support the potential modulation of children’s phenotype by maternal cardiometabolic conditions in pregnancies without medical complications.

## Introduction

Glucose and serum lipid concentrations, along with arterial blood pressure (BP) levels, are recognized indicators of cardiometabolic risk^1^ and evidence linking alterations in these indicators to adverse perinatal outcomes has been documented^2–4^. Maternal hyperglycemia has been consistently associated with higher birth weight^2,5^ and a higher risk of macrosomia^6^. According to Pedersen’s hypothesis, maternal hyperglycemia leads to an excessive flow of glucose to the fetus, resulting in fetal hyperinsulinemia, which promotes adipogenesis and leads to abnormal growth^2,6^. In contrast, maternal hypoglycemia has been associated with intrauterine growth restriction^2^. Furthermore, maternal hypertriglyceridemia is associated with higher birth weight^3,7,8^, and this can be explained by the increased transport of maternal fatty acids through the placenta, which serve as substrates for adipose tissue synthesis^9^.

On the other hand, elevated maternal diastolic and systolic blood pressure (DBP and SBP) levels have been associated with a reduced birth weight^4,10^.

However, few studies have explored the longitudinal association between cardiometabolic markers and fetal growth a crucial area of research given the dynamic nature of maternal physiology and metabolism during pregnancy. Furthermore, limited information is available on this topic for women from diverse ethnic backgrounds. This issue holds particular relevance for Latino American populations, where the prevalence of obesity and other cardiometabolic risk indicators during pregnancy has reached alarming levels, emphasizing the urgent need for evidence to perform adjustments in public health programs and policies^11,12^.

Our main objective was to evaluate the association of maternal glucose, triglycerides (TG), total cholesterol (TC), SBP, DBP with fetal and birth weight in Mexican pregnant women.

## Results

### Baselines characteristics

The total participants of the cohort were 966 women. We included 606 mother–child dyads fulfilling inclusion criteria. The baseline characteristics of the studied women are shown in Table 1**.** The average age of the participants was 25.1 (standard deviation (SD) 5.9) years, and their average height was 156.0 (SD 6.0) cm. Approximately half of the women had secondary school as a higher education level, and most lived in a consensual union. Almost half of the women had either overweight (pre-gestational body mass index (pgBMI): 25–29.9 kg/m^2^) or obesity (pgBMI: ≥ 30 kg/m^2^). Regarding the offspring, about half were female and the mean of birth weight was 3,116 (SD 381.5) grams (Table 2).

**Table 1:** Baseline characteristics of women in PRINCESA cohort in Mexico City, 2010–2015.

Characteristics	Mean (SD)
Age (years)	25.1 (5.9)
Height (cm)	156.0 (6.0)

	**% (n)**
**Pre-gestational BMI**	
Low weight	4.0 (25)
Normal	45.5 (279)
Overweight	34.3 (210)
Obesity	16.2 (99)
**Education**	
Elementary/no studies	12.0 (73)
Secondary	44.9 (274)
High school/technical	34.9 (213)
Bachelor’s degree	7.8 (50)
**Marital status**	
Married	21.9 (134)
Single/divorcee	26.7 (163)
Consensual union	51.4 (314)
**Parity**	
1	67.1 (375)
2	30.2 (169)
3 or more	2.7 (15)
**Exposure to tobacco (passive smoker)**	
Not exposed	39.0 (134)
Exposed	61.0 (212)
**Alcohol consumption**	
Do not use during pregnancy	89.0 (308)
At least once during pregnancy consumed alcohol	11.0 (38)
**Types of childbirth**	
Vaginal	63.0 (387)
C-section	37.0 (227)

BMI, body mass index; SD, standard deviation.

**Table 2:** Characteristics of neonates.

Characteristics	% (n)
**Sex**	
Female	48.1 (294)
Male	51.9 (317)

	Mean (SD)
**Estimated fetal weight (g)**	
Month 3	102.6 (20.3)
Month 4	178.5 (51.8)
Month 5	393.0 (115.1)
Month 6	795.7 (209.4)
Month 7	1,418.5 (301.0)
Month 8	2,214.0 (381.9)
Month 9	2,930.0 (373.3)
**Birth weight (g**)	3,115.8 (381.5)

SD, standard deviation.

### Trajectories of cardiometabolic risk indicators

**Figure 1:**
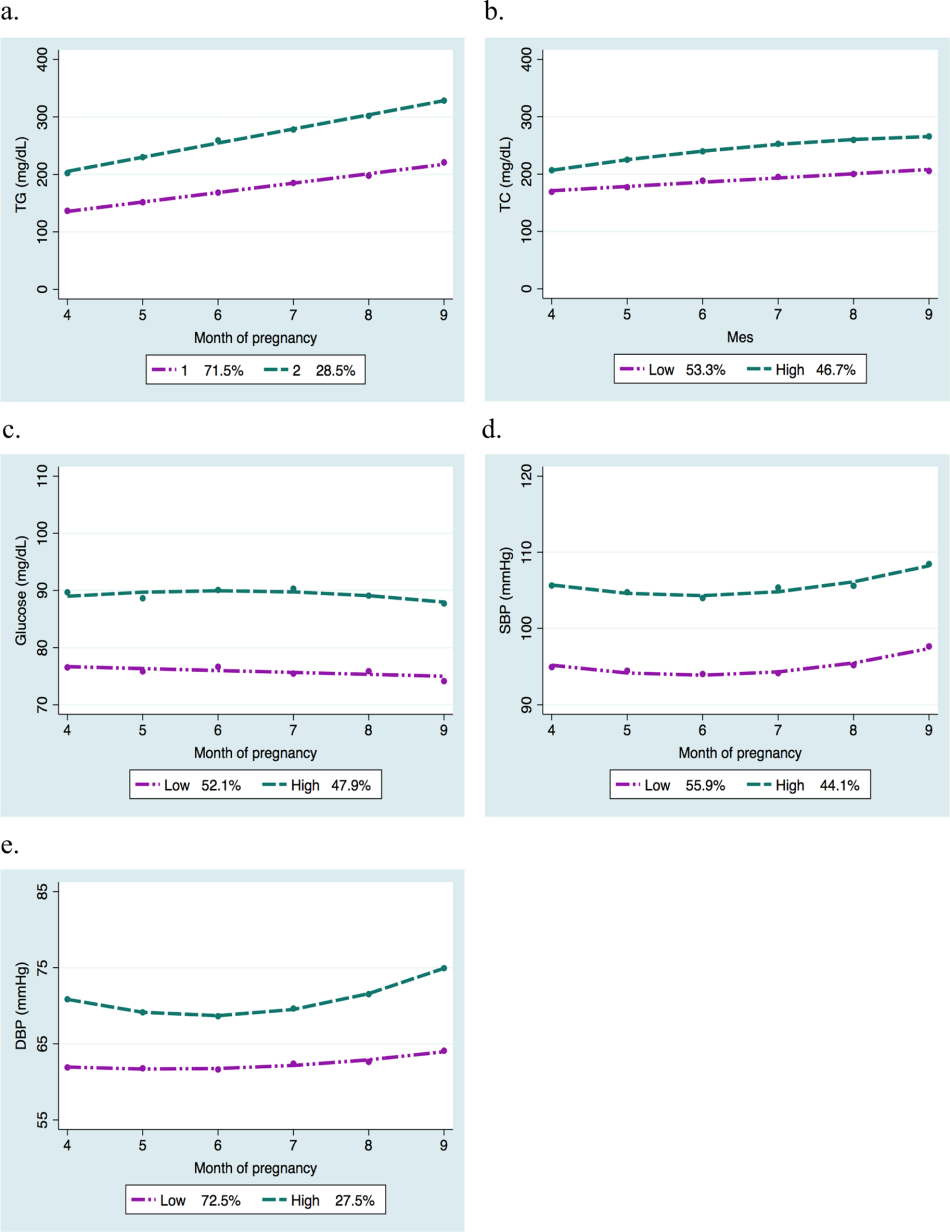
Mean values of maternal cardiometabolic markers in the high and low trajectory groups throughout pregnancy. (**a**) TG group trajectories; (**b**) TC group trajectories; (**c**) Glucose group trajectories; (**d**) SBP group trajectories; (**e**) DBP group trajectories. TG, triglycerides; TC, total cholesterol; SBP, systolic blood pressure; DBP, diastolic blood pressure. The percentages correspond to the proportion of women in each trajectory group respect the total sample.

The models showed better adjustment when considering two trajectory groups, which we referred to as “low” and “high” for maternal TG, TC, glucose, and BP during pregnancy (Fig. 1a,b,c,d,e). Within the group classified as having low trajectories, there were 325 women for TC (53.3%), 323 for glucose (52.1%), 351 for SBP (44.1%), 170 for TG (29.5%), and 163 for DBP (27.5%).

Women’s clinical characteristics in the low and high trajectory groups, were compared (Table 3). Women in the high trajectories for TG, glucose and DBP during pregnancy were older than those following low trajectories. Women in the high trajectories of TG, glucose and SBP had higher pgBMI. No significant differences were observed between the trajectory groups regarding GWG. Women in the high trajectories of lipids and BP had a shorter gestational age at term.

**Table 3:** Maternal characteristics according to cardiometabolic markers groups trajectories.

Group trajectories	Age (years) Mean (SD)	*p*-value	pgBMI Mean (SD)	*p*-value	GWG (kg) Mean (SD)	*p*-value	GE at birth Mean (SD)	*p*-value
Low TG	24.7 (5.9)	< 0.001	25.6 (5.5)	0.04	9.11 (5.7)	0.5	39.5 (1.5)	< 0.001
High TG	26.2 (6.2)		26.0 (4.5)		8.9 (5.3)		39.2 (1.2)	
Low TC	25.0 (5.8)	0.06	26.4 (5.5)	< 0.001	9.2	0.1	39.5 (1.5)	< 0.001
High TC	26.0 (6.2)		24.9 (4.8)		8.9		39.2 (1.3)	
Low glucose	24.4 (5.7)	< 0.001	25.2 (4.8)	< 0.001	9.1 (5.3)	0.5	39.4 (1.4)	0.3
High glucose	26.0 (6.2)		26.3 (5.6)		9.0 (5.9)		39.4 (1.4)	
Low SBP	24.0 (5.7)	< 0.001	24.3 (4.8)	< 0.001	9.1 (5.1)	0.9	39.5 (1.4)	< 0.001
High SBP	26.5 (6.0)		27.6 (5.3)		9.1 (6.1)		39.3 (1.4)	
Low DBP	24.5 (5.9)	< 0.001	24.8 (4.8)	0.05	8.9 (5.1)	0.02	39.5 (1.4)	< 0.001
High DBP	26.9 (5.9)		28.2 (5.7)		9.5 (6.8)		39.2 (1.3)	

TG, triglycerides; TC, total cholesterol; SBP, systolic blood pressure; DBP, diastolic blood pressure; pgBMI, pre-pregestational BMI; GWG, gestational weight gain; GE, gestational age.

### Association between trajectories of cardiometabolic indicators with estimated fetal weight and birth weight

The results of the adjusted mixed models are presented in Table 4**.** For all models, the reference category was the group of women who followed a low trajectory during pregnancy.

**Table 4:** Adjusted mixed models with intercept centered in sixth month of pregnancy: estimated fetal weight as an outcome and cardiometabolic risk trajectories as exposures.

**Cardiometabolic marker group trajectories**	$\widehat{\beta }$	*p*	95%CI
**TG**	48.24	0.021	7.2, 89.7
Interaction TG*month	24.00	< 0.001	12.9, 35.3
Interaction TG*month^2^	0.77	0.813	− 5.6, 7.2
**TC**	41.26	0.029	4.3, 78.2
Interaction TC*month	0.71	0.886	− 9.1, 10.5
Interaction TC* month^2^	− 3.87	0.168	− 9.4, 1.6
**Glucose**	− 16.39	0.392	− 53.9, 21.2
Interaction glucose*month	− 13.10	0.011	− 23.1,− 3.0
Interaction glucose* month^2^	− 1.19	0.999	− 6.8, 4.4
**SBP**	− 42.40	0.039	− 82.7,− 2.1
Interaction SBP* month	− 3.46	0.508	− 13.71, 6.8
Interaction SBP* month^2^	2.48	0.331	− 2.9, 8.6
**DBP**	− 50.35	0.025	− 94.2,− 6.4
Interaction DBP*month	− 7.90	0.173	− 19.3, 3.5
Interaction DBP*month^2^	1.84	0.567	− 4.5, 8.1

The reference category was the group of women with low trajectory during pregnancy. The $\widehat{\beta }$ coefficients showed are for the high group trajectories categories. All models were adjusted by maternal weight, age, height, education, parity, marital status and fetal sex.

TG, triglycerides; TC, total cholesterol; SBP, systolic blood pressure; DBP, diastolic blood pressure.

^2^Quadratic term of month.

**Figure 2:**
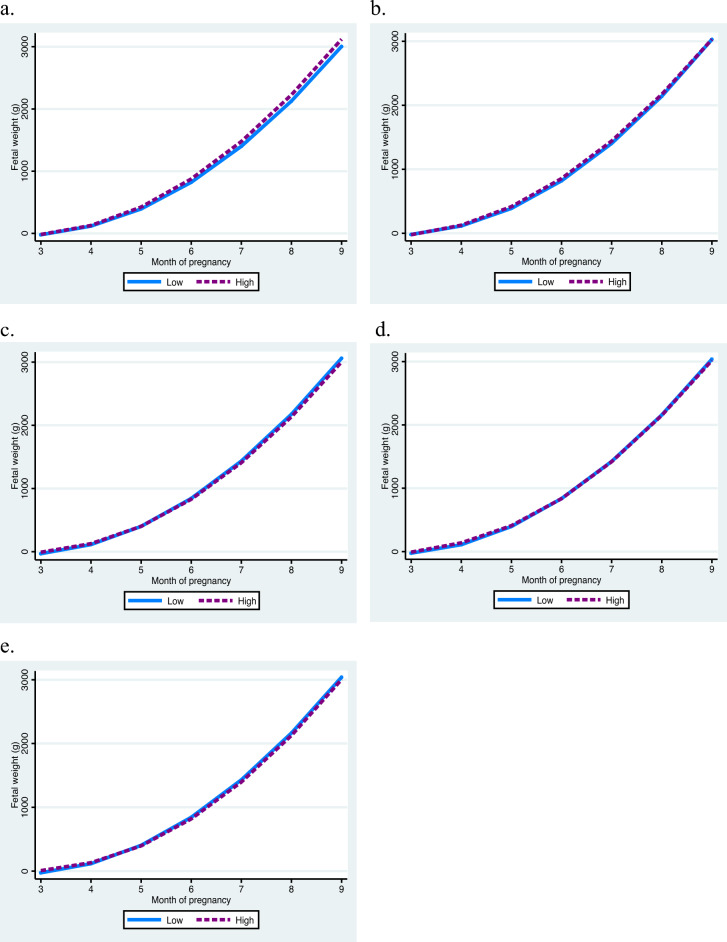
Estimated fetal weight trajectories according to maternal cardiometabolic group trajectories. (**a**) Estimated fetal weight according to maternal TG group trajectories; (**b**) Estimated fetal weight according to maternal TC group trajectories; (**c**) Estimated fetal weight according to maternal glucose group trajectories; (**d**) Estimated fetal weight according to maternal SBP group trajectories; (**e**) Estimated fetal weight according to maternal DBP group trajectories. TG, triglycerides; TC, total cholesterol; SBP, systolic blood pressure; DBP, diastolic blood pressure.

Fetuses of women classified as high TG trajectory were heavier in the sixth month of pregnancy ($\widehat{\beta }$=48.24 g) and had more weight increase over gestation ($\widehat{\beta }$=24.00 g for interaction with month) compared with fetuses from women in low TG trajectory (Fig. 2a). Similarly, women in high TC trajectory had fetuses with higher weight in the sixth month of pregnancy ($\widehat{\beta }$=41.26 g). This effect on estimated fetal weight was lost in subsequent months (Fig. 2b).

Fetuses from women classified in the high trajectory of glucose had lower weight ($\widehat{\beta }$= − 13.10 g for interaction between glucose and month) in the sixth month of gestation (Fig. 2c). Similarly, fetuses whose mothers had high SBP and high DBP trajectories weighed less ($\widehat{\beta }$= − 42.4 g and $\widehat{\beta }$= − 50.35 g, respectively) than those whose mothers were in the low trajectories. However, the effect on estimated fetal weight gain after this month was not statistically significant (Fig. 2d,e).

TC and SBP group trajectories did not shown a significant effect on birth weight in bivariate models and did not change the coefficient estimations in the adjusted models; therefore, we excluded these groups from final model. Neonates from women with high TG trajectory had higher birth weight than those of women with low trajectory ($\widehat{\beta }$=89.08 g, Table 5). Opposite to this, newborns from mothers with high DBP trajectory had less birth weight than those from mothers in low trajectory ($\widehat{\beta }$= − 101.48 g, Table 5). Glucose, TC, and SBP trajectories were not associated with birth weight.

**Table 5:** Lineal regression model with birth weight as outcome and TG and glucose trajectories as exposures.

Cardiometabolic marker group trajectories	n = 553		95%CI
	$\widehat{\beta }$	p	
High TG trajectory	89.08	0.011	20.8, 157.4
High glucose trajectory	22.70	0.479	− 40.2, 85.6
High DBP trajectory	− 101.48	0.008	− 176.5,− 26.4

The reference category was the group of women with low trajectory during pregnancy. All models were adjusted by maternal weight, maternal age, maternal height, education, parity, marital status, gestational age at birth, and neonates’ gender.

TG, triglycerides; TC, total cholesterol; DBP, diastolic blood pressure.

## Discussion

Women with normal pregnancies, defined as those with a gestational age at birth greater than 37 weeks and without any medical or obstetrical complications, were classified into two groups based on their trajectories of cardiometabolic markers. These groups were named as high and low trajectories, reflecting that we may distinguish women and fetuses with differential exposition to metabolite concentrations and BP values. Several associations between these groups and fetal effects were found. These two categories of cardiometabolic biomarkers may potentially represent a differential risk for women’s cardiometabolic health later in life. Women in high trajectories tended to be older, had higher pgBMI, and had shorter pregnancy durations. This combination, along with elevated values of cardiometabolic markers, suggests that they may represent a high-risk group. Cardiovascular disease is the leading cause of mortality for adult women and the specific contribution of risk exposition during pregnancy has not been sufficiently explored in different populations^13^.

Moreover, pregnancy triggers a compartmentalized inflammatory state required for the maintenance of gestation^14^. It has been suggested that lipid levels may modulate this state by promoting a low-grade inflammatory response^15^, potentially influencing fetal growth. Nevertheless, more evidence regarding this mechanism of damage is needed.

We identified several associated effects on fetal growth that could represent adverse environmental factors for fetal development. Increased estimated fetal weight gain and higher birth weight were associated with a high maternal TG trajectory during pregnancy. In contrast, fetuses from women with high glucose, SBP, and DBP trajectories showed reduced estimated fetal weight gain. Consequently, newborns from mothers with high DBP trajectories had a lower birth weight.

We centered the intercept in the mixed-effect model at the sixth month of pregnancy to ensure its interpretation made biological sense. The sixth month represents the beginning of the third trimester of pregnancy, in which fetal growth is more accelerated than in the previous trimesters. In this way, we could identify if, at the end of the first two trimesters, the maternal trajectories of cardiometabolic risk indicators had had an effect before the third trimester. And the use of mixed-effect models allowed us modeling the fetal growth trajectories throughout pregnancy according to the maternal group trajectories.

Our results are consistent with other studies^16–18^ describing the positive association between maternal TG and birth weight. Regarding the positive association between TG trajectories and estimated fetal weight gain, no previous studies were identified that evaluated fetal weight as an indicator of fetal growth to assess its association with maternal TG levels.

Taking into consideration Pedersen’s hypothesis, we can hypothesize that the increased availability of TG may lead to fetal metabolic programming, resulting in the accumulation of adipose tissue. This could potentially explain the higher estimated fetal weight observed in the sixth month of pregnancy and the subsequent increased estimated fetal weight gain and higher birth weight. Unfortunately, we did not measure newborn body composition to confirm this condition. Nevertheless, other studies provide support for this possibility^19,20^.

In the literature, there is limited epidemiological evidence of the association of TC level with fetal growth^16,17,21,22^. Some studies found that low concentrations of maternal TC during pregnancy were associated with lower birth weight^21^ or higher risk of small-for-gestational-age neonates^22^, while others have shown no association with birth weight^16,17^. In our study, we observed that fetuses from women with higher values of TC were heavier in the sixth month of pregnancy; however, no statistically significant differences in birth weight were found between trajectory groups. This apparent paradox can be explained by a mix of periods of rapid growth followed by deacceleration. The limited availability of TC during early pregnancy may interfere with central nervous system growth and development, which could be correlated with microcephaly and growth retardation, as it has been reported^23^. However, the impact of elevated maternal concentrations of TC on fetal growth remains unknown. Transference of cholesterol to the fetus is dependent on the concentration gradient^9^; therefore, it is plausible that several steroids, including cholesterol, have increased availability to the intrauterine compartments, resulting in placental and/or fetal effects.

Non-progressive fetal growth has been observed in fetuses of women with GDM. One study reported a reduction in estimated fetal weight among women with GDM during the 24th week of gestation, with a subsequent increase in estimated fetal weight^24^. Another study conducted in the United States observed a significant association between gestational diabetes mellitus (GDM) and an increase in estimated fetal weight from the 28th week of gestation through the end of pregnancy. Furthermore, it was found that glucose concentrations during weeks 10–14 were associated with an increase in estimated fetal weight during late gestation^25^. Another study found that women without GDM but with an increasing glucose trend during early pregnancy were associated with decreased fetal growth rates in mid-pregnancy and increased rates toward the end of pregnancy^26^. We observed a similar association: fetuses from women with a high glucose trajectory showed less estimated fetal weight increase throughout pregnancy. However, by the end of pregnancy, the differences in estimated fetal weight gain between fetuses from mothers with high or low glucose trajectories were not significant. Similarly pre-gestational type 2 diabetes increases the risk of slower growth in early pregnancy due to poor glucose control in the pre-gestational period^24,26^. This could be attributed to maternal hyperglycemia, which induces a proinflammatory and oxidative stress state in early pregnancy, temporarily inhibiting trophoblast and placental growth^27^. The same effect could be behind the negative association found between estimated fetal weight in early pregnancy and the high glucose group trajectory in the present study. In other studies, a positive association between glucose levels and birth weight has been observed^28,29^; however in our study no differences in birth weight were found between glucose groups. This could be explained by the absence of hyperglycemia in any participant and the exclusion of women developing gestational diabetes during follow-up. This could be explained by the absence of hyperglycemia in any participant and the exclusion of women developing gestational diabetes during follow-up.

With few exceptions^4^, several studies have reported a negative association between birth weight and DBP values at different stages of pregnancy^4,10,30,31^. Consistent with these findings, we found that newborns from mothers in the high DBP trajectory group had lower birth weight. This association suggests that elevated levels of maternal BP could restrict fetal growth. It has been proposed that the underlying physiological mechanism behind this association could be a form of subclinical preeclampsia, potentially causing placental damage that restricts fetal growth^10^. Numerous studies have described the effect of pgBMI on fetal growth^32–35^. However, our results suggest that maternal cardiometabolic biomarker trajectories have a direct influence on fetal growth, independently of maternal weight and height. Future research should aim to elucidate the biological pathways linking these effects.

Our findings demonstrate that the levels and trajectories of cardiometabolic health markers present in normal pregnancies are associated with disruptions in fetal growth and, potentially, fetal development.

Alterations in fetal growth caused by an adverse intrauterine environment are mediated by epigenetic mechanisms^36^. Regarding this, there is evidence that the upregulation or downregulation of certain placental microRNAs and circulating microRNAs are present in women with pathologies during pregnancy, such as preeclampsia^37^. To detect placental alterations related to abnormal fetal growth, other measurements have been proposed, such as the uterine arteries pulsatility index^38^, and counts of endothelial progenitor cells and natural killer cells^39^. However, these proposed measurements are often inaccessible in many clinical settings where prenatal care is provided.

One strength of this study is its longitudinal design, which enabled us to model monthly estimated fetal weight trajectories and maternal cardiometabolic biomarkers trajectories starting from the first trimester of pregnancy. Additionally, our study used two indicators of fetal growth and most published studies have considered only birth weight. Limitations of the study include the lack of a probabilistic sample, the homogeneity of the participants in terms of ethnicity, and their low socioeconomic and educational status. These factors may limit the generalizability of our findings to other populations. Nevertheless, our results can be extrapolated to populations with similar sociodemographic characteristics to those in Mexico.

## Conclusions

Two groups of pregnant women were identified according to their cardiometabolic markers trajectories (high and low values). These maternal groups’ trajectories were associated with adverse effects on fetal growth. Therefore, our findings support the potential modulation of children’s phenotype by maternal cardiometabolic conditions even in normal pregnancies. Maternal cardiometabolic trajectories reflect physiological dynamics during pregnancy; hence, their usefulness as clinical tools should be assessed as markers of morbidity risk for both women and fetuses. The above aim is to prevent adverse outcomes for the mother–child dyad.

Furthermore, our results highlight the relevance of short- and medium-term clinical follow-up of women after pregnancy, even if they have not developed complications, especially in those with higher cardiometabolic biomarkers and other conditions such as obesity.

## Methods

### Design and population

PRINCESA cohort (Pregnancy Research on Inflammation, Nutrition and City Environments: Systematic Analyses) was conducted at the *Hospital Materno Infantil Inguarán* in Mexico City from 2010 to 2015. This hospital provides perinatal care to the low socioeconomic status population. The participants were recruited from week 10 of pregnancy and followed until delivery with monthly assessments. The inclusion criteria for the present study were women aged 18 to 45 years and to have attended at least three prenatal check-ups. Only women with full-term, higher than 38 weeks of gestation, and normal evolution of pregnancy were included. Women with complications such as gestational diabetes mellitus (GDM) (confirmed by oral glucose tolerance) and preeclampsia and those who had preterm delivery were excluded from this analysis. All participants signed an informed consent letter, and the study was approved by the IRBs from UNAM, the University of Michigan and the Ministry of Health of Mexico City (register 102-2009 and 101/010/08/09). The research was conducted following the Declaration of Helsinki.

### TG, TC, glucose, SBP and DBP

After a minimum of 8 h of fasting, a venous blood sample was collected during each follow-up visit. The mean blood samples number for participant was 5.0 (SD 0.9), ranging from 3 to 7. The serum was separated through centrifugation and stored at − 80 °C until processing. Serum levels of TG, TC and glucose were quantified using the Adaltis automated system and SpinReact reagents (Spin React, Clinical Diagnostics, Paris, France). BP measurements were taken by pre-standardized operators using aneroid sphygmomanometers following the protocol of the American Heart Association^40^.

### Fetal weight and birth weight

Fetal weight was estimated using ultrasound with a GE Voluson E6 device, which considers the Hadlock formula^41^. This formula takes into account measurements of biparietal diameter, head circumference, abdominal circumference, and femur length. To identify outliers for this variable, a threshold of ± 3 standard deviations (SD) was applied. Birth weight was obtained from the clinical chart at the hospital.

### Covariates

Data about age, education (no studies/elementary school, secondary school, high school, and university), marital status (single/divorced; married; consensual union), and parity (1 pregnancy; 2 pregnancies; 3 pregnancies; more than 3 pregnancies) of the mothers were obtained from the questionnaire that was applied in the first visit at the hospital.

Gestational age at the initial visit was determined by first assessing the date of the last menstrual period and subsequently confirmed through a gestational ultrasound examination conducted before the 14th week. Although gestational age was recorded as the number of weeks, the assessments were conducted monthly. Therefore, pregnancy weeks were then categorized into months according to the following criteria: month 2 from weeks 5 to 8.6; month 3 spanned weeks 9 to 13.6; month 4 covered weeks 14 to 17.6; month 5 included weeks 18 to 22.6; month 6 consisted of weeks 23 to 27.6; month 7 extended from weeks 28 to 31.6; month 8 encapsulated weeks 32 to 35.6; and month 9 comprised weeks 36 to 40.6^42^.

Maternal weight was measured in each visit with a TANITA weight scale with a precision of 0.01 kg. Dietary intake assessed with a multiple-step 24-h dietary recall. The daily intake of energy and macronutrient was estimated using the food composition tables complied by the National Institute of Public Health^43^. Fetal sex was obtained from the clinical records where the neonates where born.

### Statistical analysis

Descriptive analyzes were performed to characterize the sample. For continuous variables, means and standard deviation (SD) were calculated. For categorical variables, absolute and relative frequencies were estimated.

TG, TC, glucose, SBP and DBP trajectories were identified using group-based trajectory models (GBTM). These models identify groups of individuals with similar values for the analyzed variable over time^44,45^. To choose the model with the best fit we compared the BIC (*Bayesian Information Criterion*) value between models; in addition, we considered that the groups in each trajectory were the best balanced (i.e., avoid small size groups). Descriptive analyzes were done to identify maternal characteristics according to the cardiometabolic group-based trajectory.

The mean estimated fetal weight was calculated and plotted against the gestational month to determine whether the fetal weight trajectory exhibited a linear, quadratic, or cubic pattern. To choose the best model, we compared the log-likelihood of the different models.

To evaluate the association between maternal cardiometabolic trajectories and estimated fetal weight trajectory, crude and adjusted mixed models were estimated^46^. Whereas, to assess the association of cardiometabolic trajectories with birth weight, we performed ordinary linear regression models. In these models, the gestational month was the time variable, centered around the sixth month, with the objective that the interpretation of the intercept in the models had a biological sense.

The identification code of women was considered as the random intercept. Interactions of cardiometabolic trajectories with month of pregnancy were tested to differentiate the cross-sectional and longitudinal effects of cardiometabolic trajectories on estimated fetal weight.

All models were adjusted by the following covariates: maternal weight, maternal height, maternal age, education, marital status, parity, and newborns’ sex.

Given the evidence suggesting fetal sex-specific responses to environmental stress^47^, we estimated male and female fetal weight trajectories based on each maternal cardiometabolic group trajectory. Differences between female and male trajectories were evaluated by two sample T tests, contrasting the predicted values for the models by fetal sex. No significant differences were found (data no shown in tables). Therefore, fetal sex was considered as covariable in the final models.

We adjusted models by maternal weight and height instead of gestational weight gain (GWG) because the last variable (categorized or continuous) does not consider maternal height. Physiologically, an identical gain in weight for women of different heights may have different implications. To prevent overfitting and considering the factors mentioned above, we decided not to adjust the models for pBMI.

Given the correlation between TAS and TAD, we performed separate models to assess the effect of TAS group trajectory and TAD group trajectory over estimated fetal weight. Each model was adjusted for TG, TC, glucose group trajectories, as well as the specified covariables and confounders. The effect of TG, TC, and glucose group trajectories was derived from the model with the lower β coefficients for these variables.

Regarding energy and macronutrient intake, we decided not to adjust the models by these variables because their inclusion in the models did not affect the estimation of the coefficients and considerably reduced the sample size.

## Data Availability

The datasets generated and/or analyzed during the current study are not publicly available due to participants did not explicitly consent to share their data on a public site but data is available from the corresponding author (Dr. Felipe Vadillo-Ortega) on reasonable request.
